# Supplementary material for: Deep learning-based prediction of major arrhythmic events in dilated cardiomyopathy: A proof of concept study
Source: PLoS One. 2024 Feb 29;19(2):e0297793. doi: 10.1371/journal.pone.0297793 (PMC10903812; doi:10.1371/journal.pone.0297793)
Supplement: S1 Appendix — (PDF) [file pone.0297793.s001.pdf]

Supplementary Material

S1 Appendix. Extended methods

Data preprocessing

CMR sequences

CMR images were differentiated according to the number of dimensions that characterized them. Hypervideo cine-SAx sequences were composed of 3 spatial dimensions (i.e., width, height, and slice) and 1 time dimension. There was heterogeneity between patients regarding the number of frames (temporal dimension) and the number of slices (spatial dimensions) acquired. The maximum number of frames was 30, so we augmented all the cine-SAx sequences, including “null frames”, up to 30 frames. The same process of adding “null slices” was applied to all SAx (i.e., both cine and LGE) with less than 25 slices. After preprocessing, all hypervideo cine-SAx images consisted of a 30x640x480x25 dimensional array (i.e., raw original pixels plus the “null” augmented ones).

Standard video cine-LAx sequences were composed of 2 spatial dimensions (i.e., width and height) and 1 time dimension. To obtain the same number of dimensions for each cine sequence, they were represented like single-slice hypervideos, i.e., 4-dimensional with a single level in the last one only. After that, the same preprocessing steps of the cine short axis were applied to each video. Therefore, all video cine-LAx consisted of 30x640x480x1 dimensional arrays.

Standard LGE-LAx images were composed of 2 spatial dimensions (i.e., width and height). In the same way as for cine-LAx, to obtain the same number of dimensions as LGE-LAx hyperimages, they were represented as single slice hyperimages, i.e., 3-dimensional arrays with a single level in the last dimension. After that, all LGE-LAx images were represented as 256x256x1 dimensional arrays.

Hyperimages LGE-SAx sequences were composed of 3 spatial dimensions (i.e., width, height, and slice). Therefore, “null slices” were added when necessary to obtain homogeneous hyperimages represented as 640x480x25 dimensional arrays.

Image and covariate analysis

Long short-term memory (LSTM) networks are a particular type of recurrent neural network (RNN) able to model sequencies in input and output. Thanks to LSTM architecture based on a memory cell and input, output, and forget gates, it is able to maintain complex relevant information (i.e., temporal correlation) across the sequences even for long-range dependencies.[1–3] At the same time, convolutional neural networks (CNNs) are designed to specifically model complex spatial correlations from the input data.[4–6] To process 4D and 3D cine-CMRs, we adopted ConvLSTM architectures, i.e., LSTM models adopt convolutional structures in all the internal transitions of the recurrent architecture.[7]

The final architecture proposed concatenated all four cine-CMRs in a c-1 input tensor (i.e., a multidimensional array) of shape 30x640x480x28 (time x width x height x slice), rescaled from 0-255 (image grey-level pixel-intensity range) to 0-1, and processed them with 32 2D-ConvLSTM with a 4x4 mask to a c-2 tensor of shape 640x480x32. At the same time, all four LGE-CMRs were concatenated in an l-1 tensor of shape 256x256x28. The LGE-SAx, which is originally of shape 640x480x25, was previously preprocessed by two early layers: the first, padding it to 767x512x25,

and the second by a 2x2 max-pooling mask with a 3x2 stride CNN transforming it to a tensor of shape 256x256x25.l-1 is processed by a 32 2D CNN with a 4x4 mask to an l-2 tensor of shape 256x256x32. The same padding + max-pooling processing is applied to c-2 to obtain the c-3 tensor of shape 256x256x32 as well. Next, c-3 and l-2 are simultaneously processed by the same 16 2D CNN outputting two corresponding transformed c-4 and l-3 tensors of shape 256x256x16 each, concatenated together in an m-1 tensor of shape 256x256x32 passed to further 16 2D CNNs outputting m-2 of shape 256x256x16. After flattening them in a linear tensor of shape 1048576, 8 fully connected networks encode the result in a tensor m-3 of shape 8. At the same time, the 18 baseline features f-1 (i.e., a tensor of shape 18) are shifted and scaled to be approximately in 0-1 (i.e., shift is set to zero for every continuous or Boolean covariate, while it is set to 1 for categorical covariates originally encoded with integers starting from 1; scale was set as the inverse of a fixed maximum value for each one) and processed by two consecutive sets of 8 fully connected networks producing a tensor f-2 of shape 8. Therefore, m-3 and f-2 were concatenated in an m-4 tensor of shape 16 and finally processed by two consecutive sets of 8 fully connected networks and a final single fully connected network to the o-1 output tensor of shape 1 (i.e., our)$\hat{h}_{DARP-D}(x)$). To speed up the training, taking under control the overfitting and protecting from exploding and vanishing weights, after each described layer of the network is stacked, the following layers are stacked: batch normalization, activity regularization (with both ℓ_1 and ℓ_2 regularization factors set to 0.1 for all the weights), and drop-out (set to drop 20% of weights for input layers, 70% for hidden and recurrent layers, and to 12.5%, i.e., a single neuron dropped, for the last two final layers).(28)

Survival model

Cox proportional hazard is the standard model for survival (i.e., time-to-event) analyses using individual covariate information in the estimations of the survival function. [10] In the Cox model, the hazard function $\lambda(t|X)$ represents the risk of an event at each time $t > 0$ based on the effect of the observed covariates X and under the hypotheses that the subject has survived until time t. The defined relation is based on a base risk $\lambda(t)$ supposed to be the same for all subjects and assumes individual risk modification as a multiplicative exponential term for the base risk independent of time. The hazard function is then defined as $\lambda(t|X) = \lambda(t)e^{X\beta}$. There, the term $X\beta$ is a linear combination of the individual covariates X and corresponding weights β estimated by the maximization of the Cox partial likelihood:

$$L_c(\beta) = \prod_{i \in \{i:\, E_i = 1\}} \frac{e^{\beta^T x_i}}{\sum_{j \in \Re(T_i)} e^{\beta^T x_j}}$$

where E_i is the event indicator for subject i (i.e., $E_i = 1$ if the event occurred, $E_i = 0$ if the event did not occur, or the subject was right censored), T_i is the last known time for subject i (i.e., the event or censored time), and $\Re$(t) is the risk set at time t (i.e., the subjects are still event-free at time t and so at risk at t).

Calling $h(x)$ the log-hazard function represented by $h(x) = \beta^T x$ in the Cox model, nonlinear extension to this model based on deep-learning architectures to estimate the individual log-hazard function is already present in the literature.[11–13] In 1995, Faraggi and Simon proposed a method for nonlinear survival models using a simple feed-forwards neural network with a single linear output layer $\hat{h}_\theta$(x) estimating $h(x)$ and modifying the partial likelihood accordingly.[12] In 2018, with

DeepSurv, Katzman et al. extended the Faraggi-Simon method by applying a deeper network and modern deep learning techniques to design and optimize the network (i.e., its weights θs) minimizing an objective loss function defined as the average of the modified Cox negative log partial likelihood including an ℓ_2 regularization component.

$$L_{DS}(\theta) = -\frac{1}{N_{E=1}} \sum_{i \in \{i: E_i = 1\}} (\hat{h}_\theta(x_i) - log \sum_{j \in \Re(T_i)} e^{\hat{h}_\theta(x_j)})$$

Here, $N_{E=1}$ is the number of subjects at risk.[13] There, the network structure was a sequential design of fully connected layers followed by a dropout layer to address overfitting. Inputs remain baseline covariates only.

In April 2022, Popescu et al. proposed SSCAR, a survival deep learning model to estimate individual patient times to arrhythmic SCD (SCDA).[11] In their model, they embedded both MRI 3D images (LGE-CMR only, two channels for 12 slices of interpolated 64x64 images to an array input size of 64x64x12x2, i.e., width, height, slice, and channel correspondingly) and clinical covariates. They implemented a two independent-branch network: the first is an encoder-decoder that compresses images well, and the second is a sequential fully connected network for the baseline covariates. Each branch outputs in a two-neuron layer estimating the parameters μ and σ for the probability distribution of time-to-SCDA, merging them in the final model output as a per-patient individual cause-specific survival curve based on the individual log-logistic distribution:

$$S_i(t; \mu_i, \sigma_i) = \frac{1}{1+e^{\frac{\log(t)-\mu_i}{\sigma_i}}}.$$

We propose a per-patient survival model powered by modern deep-learning techniques able to process uncompressed noninterpolated raw time-dependent 4D (cine-SAx) hypervideos, 3D (cine-LAx) videos, 3D (LGE-SAx) hyperimages, 2D (LGE-LAx) images, and baseline patient covariates in a unique heterogeneous network to directly estimate the individual nonlinear log-hazard function $h(x)$ as $\widehat{h_\theta}(x)$, i.e., the single-neuron last layer output of our neural network.

References

1. Graves A. Generating Sequences With Recurrent Neural Networks. arXiv; 2014. doi:10.48550/arXiv.1308.0850

2. Pascanu R, Mikolov T, Bengio Y. On the difficulty of training Recurrent Neural Networks. arXiv; 2013. doi:10.48550/arXiv.1211.5063

3. Hochreiter S, Schmidhuber J. Long Short-Term Memory. Neural Comput. 1997;9: 1735–1780. doi:10.1162/neco.1997.9.8.1735

4. Neural network recognizer for hand-written zip code digits | Proceedings of the 1st International Conference on Neural Information Processing Systems. [cited 18 Dec 2022]. Available: https://dl.acm.org/doi/10.5555/2969735.2969773

5. LeCun Y, Bengio Y, Hinton G. Deep learning. Nature. 2015;521: 436–444. doi:10.1038/nature14539

6. Alom MZ, Taha TM, Yakopcic C, Westberg S, Sidike P, Nasrin MS, et al. A State-of-the-Art Survey on Deep Learning Theory and Architectures. Electronics. 2019;8: 292. doi:10.3390/electronics8030292

7. Shi X, Chen Z, Wang H, Yeung D-Y, Wong W, Woo W. Convolutional LSTM Network: A Machine Learning Approach for Precipitation Nowcasting. arXiv; 2015. doi:10.48550/arXiv.1506.04214

8. Srivastava N, Hinton G, Krizhevsky A, Sutskever I, Salakhutdinov R. Dropout: A Simple Way to Prevent Neural Networks from Overfitting. Journal of Machine Learning Research. 2014;15: 1929–1958.

9. Ioffe S, Szegedy C. Batch Normalization: Accelerating Deep Network Training by Reducing Internal Covariate Shift. arXiv; 2015. doi:10.48550/arXiv.1502.03167

10. Cox DR. Regression Models and Life-Tables. Journal of the Royal Statistical Society Series B (Methodological). 1972;34: 187–220.

11. Popescu DM, Shade JK, Lai C, Aronis KN, Ouyang D, Moorthy MV, et al. Arrhythmic sudden death survival prediction using deep learning analysis of scarring in the heart. Nat Cardiovasc Res. 2022;1: 334–343. doi:10.1038/s44161-022-00041-9

12. Faraggi D, Simon R. A neural network model for survival data. Statistics in Medicine. 1995;14: 73–82. doi:10.1002/sim.4780140108

13. DeepSurv: personalized treatment recommender system using a Cox proportional hazards deep neural network | BMC Medical Research Methodology | Full Text. [cited 22 Nov 2022]. Available: https://bmcmedresmethodol.biomedcentral.com/articles/10.1186/s12874-018-0482-1
