# Supplementary material for: Deep learning-based prediction of major arrhythmic events in dilated cardiomyopathy: A proof of concept study
Source: PLoS One. 2024 Feb 29;19(2):e0297793. doi: 10.1371/journal.pone.0297793 (PMC10903812; doi:10.1371/journal.pone.0297793)
Supplement: S1 Text — (PDF) [file pone.0297793.s002.pdf]

Supplementary Material

S1 Text. Technical aspects

Models were trained on a Ubuntu 22.04 LTS system without desktop support via ssh connection hosted on a Intel® Xeon® E2286M 16-CPUs @5.0 GHz equipped with 134.7 GB RAM augmented with 1.4 TB of m.2 SSD dedicated system Swap.

With the reported hardware, and considering raw uncompressed in-memory patient data were 4.5 GB/patient (w/ approximately 3.7 GB for cine-SAx alone), optimal found configuration consisted in passing patient data to the training procedure in pre-saved random batches of 6 patients each one, including 4+ events per batch to guarantee non trivial risk sets within batches.

For the first training stage we passed to the fitting procedure 32 batches each epoch, and 16 for validation. In training performances where estimated as the average of the batches performances, in the final performance estimation, all the train, validation, train+validation and test measures were evaluated globally on the whole sets at same time. Pre-saved batches were loaded on-the-fly by by generator functions into the training procedure directly.

With the reported configuration, each epoch of training takes approximately 8.5 hours for the training vs validation stage (i.e., 32 6-patients batches for training and 16 for performance monitoring on the validation set), and 12.5 hours for the training+validation vs test stage. Peak memory usage reached 134.7 GB RAM + 187 GB Swap. Prediction time was stable at approximately 40 seconds/prediction + 10 seconds/patient, using less than 16 GB RAM per patient (i.e., up to RAM availability, multi-patient predictions are possible and useful to reduce model loading time, and so overall prediction time).
