# Supplementary material for: Deep learning-based prediction of major arrhythmic events in dilated cardiomyopathy: A proof of concept study
Source: PLoS One. 2024 Feb 29;19(2):e0297793. doi: 10.1371/journal.pone.0297793 (PMC10903812; doi:10.1371/journal.pone.0297793)
Supplement: S1 Fig — Receiver operator characteristic curves (ROC) for years 1, 2, 3, 5 and 8 for the internal validation and test sets, with the respective areas under the curve (AUROC). Predicted outcomes are based on the estimated survival probability at the respective time points as computed from the survival probability function. (PDF) [file pone.0297793.s003.pdf]

Supplementary Material

S1 Fig. Survival study of major arrhythmic events at different time point

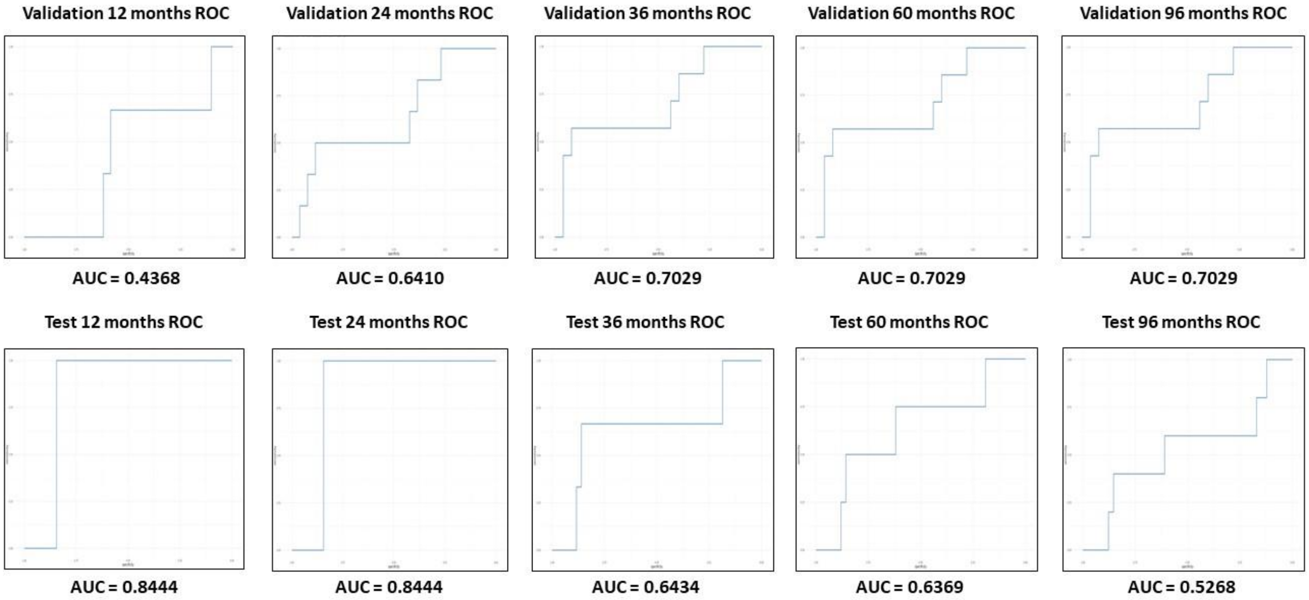

Receiver operator characteristic curves (ROC) for years 1, 2, 3, 5 and 8 for the internal validation and test sets, with the respective areas under the curve (AUROC). Predicted outcomes are based on the estimated survival probability at the respective time points as computed from the survival probability function.
