# Supplementary material for: Deep learning-based prediction of major arrhythmic events in dilated cardiomyopathy: A proof of concept study
Source: PLoS One. 2024 Feb 29;19(2):e0297793. doi: 10.1371/journal.pone.0297793 (PMC10903812; doi:10.1371/journal.pone.0297793)
Supplement: S1 Table — (PDF) [file pone.0297793.s004.pdf]

Supplementary Material

S1 Table. Baseline and CMR characteristics and follow-up.

**Supplementary Table 1**						
**Variable**	**Overall**<br>**(n = 154)**	**Train**<br>**(n = 76)**	**Validation**<br>**(n = 32)**	**Train + Validation**<br>**(n = 108)**	**Test**<br>**(n = 46)**	**p-value**
**Medication use**						
ACE inhibitor, n (%)	123(84)	62(90)	24(75)	86(85)	37(82)	0.147
ARB, n (%)	14(10)	4(6)	5(16)	9(9)	5(11)	0.283
B-blocker, n (%)	138(94)	66(96)	28(87)	94(93)	44(98)	0.126
CCB						0.673
Non-dihydropyridine, n (%)	2(1)	1(1)	1(3)	2(2)	-	
Dihydropyridine, n (%)	7(5)	2(3)	2(6)	4(4)	3(7)	
MRA, n (%)	81(55)	34(49)	22(69)	57(56)	25(56)	0.107
Sacubitril, n (%)	3(2)	1(1)	1(3)	2(2)	1(2)	0.860
Loop diuretics, n (%)	102(70)	48(70)	27(84)	75(74)	27(60)	0.071
Digoxin, n (%)	13(9)	3(4)	6(19)	9(9)	4(9)	0.058
Amiodarone, n (%)	42(29)	17(25)	13(41)	30(30)	12(27)	0.238
Ivabradine, n (%)	8(5)	2(3)	4(12)	6(6)	2(4)	0.138

Lipid-lowering, n (%)	40(27)	20(29)	7(22)	27(27)	13(29)	0.730
**CMR measurements**						
LVEDVi, ml/m^2	139(112-171)	135(113-174)	145(119-190)	135(114-182)	145(109-170)	0.213
LVESVi, ml/m^2	97(79-132)	95(70-138)	99(85-152)	95(79-140)	102(80-119)	0.211
LVEF, %	27(20-35)	26(20-38)	25(18-32)	26(19-34)	27(22-35)	0.509
RVEDVi, ml/m^2	56(48-80)	59(47-79)	59(47-86)	59(48-80)	56(48-77)	0.221
RVVTSi, ml/m^2	30(20-40)	28(19-42)	32(20-45)	30(19-43)	31(22-36)	0.089
RVEF, %	50(43-59)	52(46-60)	46(37-59)	51(42-60)	50(45-57)	0.101
**Follow-up**						
Median time, y	6.0(3.5-8.8)	7.4(3.8-9.9)	4.9(2.2-7.3)	6.3(3.7-9.2)	5.2(2.6-8.4)	0.752
CRT, n (%)	26(19)	14(20)	6(23)	20(20)	6(15)	0.761
ICD, n (%)	70(45)	36(47)	13(41)	49(45)	21(46)	0.813
All cause death, n (%)	12(8)	5(7)	3(9)	8(7)	4(9)	0.602
VAD implantation, n (%)	1(1)	-	1(1)	1(1)	-	-
Heart transplant, n (%)	12(8)	8(10)	-	8(7)	4(9)	0.170
HF hospitalization, n (%)	36(23)	15(20)	12(37)	27(25)	9(20)	0.105

1, n (%)	21(14)	11(14)	6(19)	17(16)	4(9)	
2, n (%)	7(4)	3(4)	2(6)	5(5)	2(4)	
3+, n (%)	7(4)	-	4(12)	4(4)	3(6)	
MAE, n (%)	20(16)	7(9)	7(22)	14(13)	6(13)	0.090

ACE: angiotensin-converting enzyme; ARB: angiotensin receptor blocker; CAD: coronary artery disease; CCB: calcium channel blockers CMP: cardiomyopathy; COPD: chronic obstructive pulmonary disease; CRT: cardiac resynchronization therapy; DCM: dilated cardiomyopathy; ECG: electrocardiogram; HF: heart failure; ICD: implantable cardioverter defibrillator; LBBB: left bundle branch block; LVEDVi: left ventricular end-diastolic volume index; LVESVi: left ventricular end-systolic volume index; LVEF: left ventricular ejection fraction; MAE: major arrhythmic event; MRA: mineralcorticoid receptor antagonist; NYHA: New York Heart Association; RVEDVi: right ventricular end-diastolic volume index; RVESVi: right ventricular end-systolic volume index; RVEF: right ventricular ejection fraction; SCD: sudden cardiac death; VAD: ventricular assistant device. Differences between "train", "validation" and "test" groups were assessed using the Mann-Whitney test for continuous variables and the Pearson chi square test of Fisher exact test for categorical variables. P values < 0.05 were considered statistically significant.
