# Supplementary material for: Deep learning-based prediction of major arrhythmic events in dilated cardiomyopathy: A proof of concept study
Source: PLoS One. 2024 Feb 29;19(2):e0297793. doi: 10.1371/journal.pone.0297793 (PMC10903812; doi:10.1371/journal.pone.0297793)
Supplement: Attachment — Submitted filename: Response to reviewers.docx [file pone.0297793.s005.docx]

Mattia Corianò, MD
University of Padova, Department of Cardiac, Thoracic, Vascular Sciences and Public Health, Padova, Italy
Via Niccolò Giustiniani 2
Italy, Padua, 35131
mattia.coriano@studenti.unipd.it
24 December 2023

To:

Vincenzo Lionetti

Plos One Academic Editor

Subject: Response to Reviewers for Manuscript “Deep learning-based prediction of major arrhythmic events in dilated cardiomyopathy”.

Dear Editor,

I hope this letter finds you well. I am writing to express my sincere gratitude for the thoughtful and constructive feedback provided by you and the reviewers on my manuscript. The insightful comments have significantly contributed to the improvement of the manuscript.

I appreciate the time and effort the reviewers invested in reviewing my work. Their feedback has been invaluable, and I have carefully considered each comment in the revision process. In this response, I have addressed each comment and made the necessary revisions to enhance the clarity, validity, and overall quality of the manuscript.

Below, I outline the major comments raised and detail my responses:

**Editor:** advised highlighting major limitations of the study and emphasizing the proof-of-concept nature of the research in the title.

We appreciate the Editor's insightful feedback on emphasizing the major limitations of our study and underlining the proof-of-concept nature of the research.

In accordance with this constructive advice, we have revised the title of our manuscript. Additionally, we have expanded the discussion in the manuscript to provide a more thorough explanation of the limitations, particularly focusing on the reduced sample size. Changes are at rows 334-343 and are highlighted in red.

These changes aim to provide a clearer context for readers regarding the exploratory nature of our research and the associated constraints. We believe that addressing these aspects will enhance the transparency and interpretation of our findings.

We sincerely thank the Editor for guiding us in improving the clarity and transparency of our manuscript.

**Reviewer #1** provided valuable feedback, suggesting a less technical description of the methods. Moreover, he recommended us to perform a validation of the model by increasing the cohort of patients and performing a multicentre work.

In response to this constructive feedback, we have revised the methods section of the manuscript to offer a more accessible and concise overview. In particular, we have made adjustments to the following subparts: data preparation [106]; CMR images [109-115]; Images and covariates analysis [132 – 142]; Survival model [144 – 149]. The original, more detailed, technical description was added in the Supplementary Material. All changes are highlighted in yellow.

We thank reviewer #1 for the recommendation to increase the performance of our model. We have planned a retraining-test process of the model with data from a multicentre CMR registry.

**Reviewer #2** did not suggest any changes and provided positive feedback on the manuscript.

We sincerely appreciate the time and effort invested by Reviewer #2 in reviewing our manuscript. We are grateful for the positive feedback and are pleased to hear that the content met the expectations. Your encouragement motivates us to continue our efforts in advancing this research.

**Reviewer #3** raised questions about whether concomitant causes of sudden cardiac death were considered, particularly those with no impact on CMR. Additionally, there was a query on how concomitant hidden diagnoses, such as Brugada syndrome or catecholaminergic ventricular tachycardia, were excluded, suggesting an expansion of this topic with references. Further clarifications were sought on the concept of "optimal medical therapy," differences in arrhythmias monitoring between subcutaneous and transvenous devices, and the role of a loop recorder in the patient population.

We sincerely appreciate the insightful inquiries raised by Reviewer #3. In response to these constructive comments, we have carefully revised the "Limitation" section of the manuscript. Specifically, we now explicitly state that we investigated the absence of channelopathies by reviewing anamnestic reports. However, it should be noted that no other diagnostic tests were reported to have been performed to exclude these forms of channelopathies, and this potential bias could have influenced the results.

Furthermore, we have provided a more detailed explanation of the concept of "optimal medical therapy" in accordance with the recommendations outlined in the ESC HF guidelines (2021).

Regarding the subcutaneous implantable cardioverter-defibrillator (S-ICD), we highly value this feedback. It is a crucial aspect that should be considered in future studies, especially concerning the differences in endpoint incidence based on the inclusion of antitachycardia pacing therapy. This information can significantly impact the discrimination ability of a deep learning model.

As for the role of a loop recorder in our study, we did not identify anamnestic data regarding this information during the review of anamnestic reports. We acknowledge the importance of this aspect and will consider it in our future investigations.

We express our gratitude for the valuable feedback provided, which has significantly contributed to the refinement of our manuscript.

The relevant changes can be found in rows 346 – 351, 369 – 381, 387 – 396, and are highlighted in green. All suggested references were cited.

I believe that the revisions have effectively addressed the concerns raised by the reviewers, resulting in a manuscript that is stronger and more robust. I am confident that these changes will contribute positively to the overall quality and impact of the paper.

I would like to express my appreciation for the opportunity to revise and resubmit my manuscript to Plos One. I look forward to hearing the final decision and appreciate your consideration of my work.

Thank you once again for your time and for overseeing the review process.

Sincerely,

Mattia Corianò, MD
